# P4HA1 Mediates Hypoxia-Induced Invasion in Human Pancreatic Cancer Organoids

**DOI:** 10.1158/2767-9764.CRC-24-0025

**Published:** 2025-05-30

**Authors:** Bernat Navarro-Serer, Maria F. Wissler, Brandi K. Glover, Michael G. Lerner, Harsh H. Oza, Vania Wang, Hidur Knutsdottir, Fatemeh Shojaeian, Kathleen Noller, Saravana Gowtham Baskaran, Sarah Hughes, Alana M. Weaver, Daniel Wilentz, Oluwatobiloba Olayemi, Joel S. Bader, Elana J. Fertig, Daniele M. Gilkes, Laura D. Wood

**Affiliations:** 1 Department of Pathology, Johns Hopkins School of Medicine, Baltimore, Maryland.; 2 Department of Physics, Engineering and Astronomy, Earlham College, Richmond, Indiana.; 3 Department of Oncology, Johns Hopkins School of Medicine, Baltimore, Maryland.; 4 Department of Biochemistry and Molecular Biology, Johns Hopkins Bloomberg School of Public Health, Baltimore, Maryland.; 5 Department of Biomedical Engineering, Johns Hopkins University, Baltimore, Maryland.; 6 Department of Applied Mathematics and Statistics, Johns Hopkins University, Baltimore, Maryland.

## Abstract

Pancreatic ductal adenocarcinoma (PDAC) is a highly aggressive malignancy with dismal prognosis. PDAC develops in a hypoxic environment in which cells adapt and activate processes to allow survival under low-oxygen conditions, some of which may enhance the ability of cancer cells to invade locally or metastasize distantly. Using human PDAC organoids, we determined that hypoxia consistently enhanced invasion across 11 patient-derived models. Using RNA sequencing of hypoxic invasive organoids (compared with matched invasive normoxic organoids from the same patients), we identified prolyl 4-hydroxylase subunit alpha 1 (P4HA1) as a potential regulator of PDAC invasion in hypoxia. Leveraging publicly available datasets from human tissue, we determined that P4HA1 is more highly expressed in PDAC compared with normal pancreatic tissue and that high P4HA1 expression correlates with poor patient prognosis. To further interrogate the role of P4HA1 in invasion of hypoxic patient-derived organoids, we quantified invasion in organoids modified to knockdown or overexpress P4HA1, demonstrating that P4HA1 is necessary for hypoxia-enhanced invasion and sufficient to increase invasion in normoxia in PDAC organoids. Our results identify P4HA1 as a driver of PDAC organoid invasion in hypoxia.

**Significance:**

This study demonstrates that hypoxia increases invasion across a cohort of human pancreatic cancer organoids and identifies the collagen-modifying enzyme P4HA1 as a driver of hypoxia-enhanced invasion. These results characterize a molecular mechanism by which the microenvironment alters tumor cell behavior and underscore new strategies to inhibit invasion.

## Introduction

Pancreatic ductal adenocarcinoma (PDAC), one of the deadliest malignancies with a 5-year survival rate of about 10% (1), is defined by extensive local invasion and systemic metastasis. Primary PDACs are also characterized by the development of a desmoplastic stroma, which restricts cells from nutrients and oxygen, causing tumors to progress in low oxygen levels (2). In this context, PDAC cells physiologically adapt to hypoxia by adopting processes including metabolic reprogramming and induction of angiogenesis; in addition, hypoxia can affect other cellular processes such as mobility and invasion (3–5). Thus, hypoxia emerges not just as a consequence of uncontrolled proliferation and cell growth but rather as an active player in promoting tumor progression. Importantly, although PDACs are hypoxic, little is known about the contribution of hypoxia in pancreatic cancer to tumor dissemination and invasion. Thus, understanding the mechanisms by which hypoxia induces PDAC invasion may reveal new strategies to prevent metastasis.

Human patient-derived organoids (PDO) have emerged as a valuable tool to study the biology of PDAC, including processes such as tumor growth, drug resistance, and response to therapy (6–8). Importantly, PDAC PDOs can recapitulate the complex processes that are involved in tumor cell invasion. We previously used PDO models to study the molecular alterations that drive invasion, including patterns of invasion associated with driver gene mutations and clinical outcomes as well as transcriptomic programs associated with invasion (9, 10). In addition, PDOs have recently been used to study the impact of hypoxia on response to drug treatment in PDAC (11). Thus, PDAC PDOs are promising models to study the impact of hypoxia on PDAC invasion, which remains largely unexplored.

Prolyl hydroxylation is a common post-translational modification which modulates protein folding and stability (12). Collagen prolyl 4-hydroxylase subunit alpha-1 (P4HA1) is the most common isoform of the collagen prolyl 4-hydroxylases and contributes to collagen synthesis and deposition (13). Importantly, an increase in collagen production has been associated with cancer development and progression (14, 15). Additionally, P4HA1 overexpression in cancer has been linked to tumor progression. For example, in breast cancer, P4HA1 can contribute to chemoresistance by controlling cancer cell stemness in a hypoxia-inducible factor (HIF)–dependent manner (16). In lung cancer, P4HA1 is essential for migration, growth, and invasion (17). In PDAC, P4HA1 expression has been associated with stabilization of HIF1α, creating a positive feedback loop which can regulate glycolysis and other processes such as cell proliferation, chemoresistance, and stemness (18). However, little is known about the impact of P4HA1 on PDAC invasion and migration.

In this study, we interrogate the impact of hypoxia in a PDAC PDO model, identifying P4HA1 as a key mediator of tumor invasion in hypoxia.

## Materials and Methods

### Human pancreatic cancer specimen and organoid culture generation

This study was approved by the Institutional Review Board of The Johns Hopkins Hospital and conducted in accordance with recognized ethical guidelines, and written informed consent was obtained from patients prior to sample acquisition. Details of the cohort are provided in Supplementary Table S1. All patients had a histologically confirmed diagnosis of PDAC, including seven male patients and four female patients with a mean age of 62 years (range, 51–77 years). Organoids were generated from 11 primary human PDAC samples acquired from surgical resection specimens at The Johns Hopkins Hospital, as previously described (9, 10). In brief, tissues were rinsed, minced, and then digested in 8 mL of digestion media containing Dispase (Gibco, cat. #17105-041) and Collagenase Type II (Gibco, cat. #17101-015). PDAC tissue samples were digested at 37°C for 2 to 3 hours with 200 rpm shaking. The cancer cell suspension was centrifuged for 5 minutes at 1,500 rpm at 4°C, and the pellet was washed with 10 mL of wash media (DMEM/F-12, 1.25 mL primocin, 5 mL 1 mol/L HEPES, 5 mL 100× GlutaMAX, and 2.5% FBS). The cancer cell suspension was placed on a tube rack to allow large stromal pieces to precipitate. The supernatant containing organoid and single-cell suspensions was moved to a new tube to repeat 5 seconds of centrifugation and washed four times with wash media. In general, 20 μL of cell suspension contained 20 to 30 organoids, and this number was checked under a microscope to plate about 75 organoids per dome. Matrigel domes were plated in a 24-well plate (Avantor VWR plates, cat. #100062-896) and incubated for 30 to 60 minutes until domes were solidified. Human feeding media [high-glucose DMEM/F12 (1×, Invitrogen), 10 mmol/L HEPES, 2 mmol/L L-glutamine, 100 U/mL penicillin/streptomycin, B27 (Invitrogen, cat. #17504044), 1 mmol/L N-acetyl-L-cysteine (Sigma, cat. #A7250), Wnt3a-conditioned medium (50% v/v, RRID: CVCL_0635), R-spondin I–conditioned medium (10% v/v, Calvin Kuo, RRID: CVCL_RU09), 10 nmol/L gastrin (Sigma, cat. #G9020), and 10 mmol/L nicotinamide (Sigma, cat. #N3376)] were added and changed every 3 to 4 days. Growth of PDO cultures is usually observed within the first 2 weeks. If PDOs did not significantly expand in that timeframe, the culture was discarded.

### Matrigel to collagen I transfer

Following culture expansion, the impact of hypoxia on invasion was assessed by embedding PDAC organoids into collagen I gels for time-lapse imaging. Organoids cultured in Matrigel were collected by pipetting up and down to break the domes in 10 mL of cold wash media. Organoids in Matrigel were pelleted by centrifugation at 1,500 rpm, media were removed, and the pellet was resuspended in 0.5 to 1 mL of cold cell recovery solution (Corning, cat. #CLS354253) and incubated in ice for 5 to 10 minutes. Organoids were pelleted to check for depolymerization of Matrigel. This was repeated if Matrigel did not totally depolymerize. Collagen I gel was prepared as previously described (8, 9). Collagen I was mixed with 10× DMEM and 1N NaOH to result in 3.34 mg/mL collagen (Corning, collagen I, rat, cat. #354236). The collagen solution was incubated in ice for 10 to 20 minutes (batch-dependent) until collagen fiber formation was initiated. The collagen gel was then mixed with an organoid pellet and plated on a 24-well plate (Avantor VWR plates, cat. #10062-896) and incubated at 37°C for 60 minutes to allow polymerization and solidification before low-growth media were added [basic media (DMEM/F12, 1.25 mL primocin, 5 mL 1 mol/L HEPES, and 5 mL 100× GlutaMAX), 5 ng/mL EGF (Sigma, E9644-.5 mg), 5 μg/mL insulin (Gibco, cat. #12585-014), 10 ng/mL cholera toxin (Sigma, cat. #C8052-.5 mg), and 0.075% BSA (Sigma, cat. #A1595-50 mL)].

### Microscopy and time-lapse imaging

Invasion of PDAC organoids into collagen I gel was assessed by time-lapse imaging using a Nikon ECLIPSE Ti inverted microscope system (RRID: SCR_021242). PDAC organoids were embedded into collagen I, as described above. Images were collected at 30-minute (normoxia) and 60-minute (hypoxia) intervals for 3 to 4 days at 37°C and 5% CO_2_. Additional representative images of invasive and noninvasive organoids were acquired using the Nikon Ti-E inverted microscope on the final day of time lapse. All images were taken at 10× using a differential interference contrast filter.

### Organoid collection

The manual collection of individual organoids was performed on an inverted microscope (Nikon TMS 213874, RRID: SCR_020332). Prior to collection, the pancreatic organoid media were aspirated and replaced with 2 mL of PBS to prevent collagen I gel from drying. Briefly, the organoid of interest was identified and extracted from the dome by using a scalpel. Moreover, the collagen I gel surrounding the organoid of interest was gently grasped by a 1,000 μL pipette and transferred to a tube. After the collection, the tubes were snap frozen and stored at −80°C until RNA extraction [Quick-RNA Miniprep Plus kit (Zymo Research, cat. #R1057)].

### Organoid invasion analysis

Invasion impact was assessed by analyzing the percentage of organoids invading into collagen I fibers as well as organoid circularity. Invasive organoids had their borders traced using the Polygon Selections option in Fiji (RRID: SCR_002285). To analyze differences in invasiveness, the inverse circularity score, defined as 1/circularity, was calculated and plotted. Invasiveness analysis was performed only in invasive organoids, which were determined by analyzing the shape of invasive fronts. Organoids that invaded as a result of being in contact with the well plate were not considered invasive and excluded from the overall analysis. Invasive organoids showcasing mesenchymal invasion were not selected for invasiveness analysis because of their dissemination as single cells.

### IHC

For epithelial–mesenchymal transition (EMT) markers, automated staining was performed on the Leica Bond RX (Leica Biosystems, cat. #21.2821, RRID: SCR_025548) by the Johns Hopkins Tumor Microenvironment Lab. Slides were prepared using the bake and dewax protocol in which slides were incubated for 10 minutes at 60°C and 30 seconds at 72°C with dewax solution and then washed. Antigen retrieval was performed using sodium citrate buffer at pH 6 by incubating for 20 minutes at 100°C. Primary antibody for E cadherin clone 36B5 (Leica Biosystems, cat. #NCL-E-Cad, RRID: AB_442084) or vimentin clone V9 (Leica Biosystems, cat. #NCL-L-VIM-V9, RRID: AB_564055) was applied for 30 minutes at 1:100 and 1:400 dilutions, respectively, using Antibody Diluent (AR9352). Detection was performed using the Bond Polymer Refine Kit (Leica Biosystems, cat. #DS9800). The slides were counterstained with hematoxylin for 5 minutes, washed, baked, and coverslipped using EcoMount (Biocare Medical, cat. #5082832). For hydroxyproline, immunostaining was performed at the Oncology Tissue Services Core of Johns Hopkins University School of Medicine. Immunolabeling was performed on formalin‐fixed, paraffin-embedded sections on a Ventana Discovery Ultra autostainer (Roche Diagnostics, cat. #05987750001, RRID: SCR_021254). Briefly, following dewaxing and rehydration on board, epitope retrieval was performed using Ventana Ultra CC1 buffer (Roche Diagnostics, cat. #6414575001) at 96°C for 64 minutes. Primary antibody anti‐hydroxyproline (1:15 dilution; Cell Signaling Technology, cat. #73812, RRID: AB_3674746) was applied at 37°C for 60 minutes. Primary antibodies were detected using an anti-rabbit HQ detection system (Roche Diagnostics, cat. #7017936001 and 7017812001) and amplified by Discovery AMP Multimer (Roche Diagnostics, cat. #6442544001), followed by the Chromomap DAB IHC detection kit (Roche Diagnostics, cat. #5266645001), counterstaining with Mayer hematoxylin, dehydration, and mounting.

### IHC quantification

IHC slides were scanned using the NanoZoomer S360 Digital slide scanner (Hamamatsu, cat. #C13220-01, RRID: SCR_023761). Digital image analysis was performed using HALO (RRID: SCR_018350) with the Membrane Analysis module to quantify hydroxyproline staining across the entire slide. Software automatically segmented individual cells and classified staining intensity into four categories (0, 1+, 2+, and 3+) based on predefined intensity thresholds. The H-score was calculated using the formula H-score = ∑ (percentage of cells at each intensity × corresponding intensity value), generating a final score ranging from 0 to 300. Data were exported for statistical analysis to assess staining distribution and intensity differences between experimental conditions.

### Construction of P4HA1 lentivirus expression vector and stable cell lines

For P4HA1 overexpression, lentiviral particles were obtained using VectorBuilder (control: VB010000-9298rtf and overexpression: VB900004-4124sdt). For knockdown experiments, we used a control empty vector pLKO.1 (Sigma, cat. #SHC001, RRID: Addgene_52961) and two shP4HA1 constructs: shP4HA1_1 [short hairpin RNA (shRNA) TRCN0000061998] and shP4HA1_2 (shRNA TRCN0000062000). Transformation of DH5α-competent cells (Thermo Fisher Scientific, cat. #EC0112) was performed according to the protocol. Plasmid DNA was isolated using and following the Qiagen Plasmid Plus Kit (Qiagen, cat. #12943). The production of lentivirus and cell infection was performed according to the pLKO.1 lentiviral vector protocol recommended by Addgene. Briefly, the knockdown lentiviral and pLKO.1 vectors were packaged using pCMV-dR8.2 (RRID: Addgene_8455) and pCMV-VSV-G (RRID: Addgene_138479) plasmids and were transfected into 60% to 70% confluent and antibiotic-free HEK-293T cells (RRID: CVCL_0063) using Lipofectamine and Opti-MEM media (Invitrogen). The lentiviruses were harvested twice at days 4 and 5. Virus was filtered with a 0.45-μm filter and stored at −80°C. Virus was concentrated using the Lenti-X Concentrator (Takara, cat. #631231). The Lenti-X Concentrator was added to the viral supernatant (1:3 ratio) and was left to incubate overnight at 4°C. Virus and concentrator were centrifuged at 1,500 rpm for 45 minutes at 4°C. The supernatant was aspirated and the viral pellet was resuspended in DMEM/F12 containing GlutaMAX 100X, penicillin/streptomycin (10,000 U/mL), and HEPES 1 mol/L and aliquoted into 0.25 mL aliquots. Viral aliquots were stored in −20°C. One aliquot can be used for one infection.

### Lentiviral transduction of organoids

We achieved efficient lentiviral transduction in PDAC organoids by digestion of the organoids into single cells and using the protocol as described previously (9). Briefly, human PDAC organoids were first cultured in Matrigel (Corning, cat. #354236) for 3 to 10 passages with previously described organoid feeding media. Organoids from three to four domes were freed from Matrigel using the Matrigel to collagen I protocol, resuspended in 1 mL of TrypLE (Gibco, cat. #12604013), and gently agitated at 37°C for 3 to 5 minutes until organoids were separated into single cells. TrypLE was quenched by adding 9 mL wash media and tubes were centrifuged at 1,500 rpm for 5 minutes at 4°C. The supernatant was removed, and the cell pellet was resuspended with 250 μL virus and 1 μL 250× Polybrene Stock Cell suspension. The virus was transferred into a single well of a 48-well culture plate and centrifuged at 600 RCF (∼1,700 rpm) for 1 hour at room temperature. The plate was then incubated at 37°C for 1 to 6 hours. Cells were then resuspended in 1 mL of DMEM/F12 containing GlutaMAX 100X, penicillin/streptomycin (10,000 U/mL), and HEPES 1 mol/L and centrifuged at 850 rpm for 5 minutes at 4°C. The supernatant was carefully aspirated, and the pellet was resuspended with 100 to 200 μL of Matrigel and plated into a 24-well plate (50 μL/well). Matrigel domes were allowed to harden for 1 hour and were incubated with human feeding media for 2 days before starting puromycin selection. The media were changed every 3 to 4 days. To characterize the effectiveness of constructs, total RNA and protein were extracted for control, overexpression, and knockdown organoids and RT-qPCR and Western blotting was performed. To characterize the invasion of modified organoids and the impact of P4HA1 overexpression and knockdown, the organoids were transferred from Matrigel to collagen I gels and studied in invasion assays as described above.

### Cell viability assays

The cell viability of transduced organoids was detected by CellTiter-Glo 3D Cell Viability Assay (Promega, cat. #G9681) according to the manufacturer’s protocol. Briefly, organoids cultured in Matrigel were freed from the gel by gentle agitation and resuspended in cold DMEM/F12. Intact tumor organoids were spun down by differential centrifugation and re-embedded into either Matrigel or collagen I gel, depending on the desired growth condition, and human feeding media were added. Then, the organoids were placed in normoxic or hypoxic conditions and allowed to grow for indicated time and cell viability was measured.

### Western blot analyses

Proteins from lysed organoids were fractionated by SDS-PAGE and transferred to nitrocellulose membranes. Nonspecific binding sites were blocked with 5% BSA in Tris-buffered saline solution with Tween 20 [120 mmol/L Tris–HCl (pH 7.4), 150 mmol/L NaCl, and 0.05% Tween 20] for 1 hour at room temperature. Western blotting of β-actin on the same membrane was used as a loading control. The membranes were incubated with primary antibodies overnight at 4°C. The membranes were then washed with TBST three times and incubated with a horseradish peroxidase (HRP)–conjugated secondary antibody for 1 hour at room temperature. Proteins were visualized using a SuperSignal West Pico PLUS Chemiluminescent Substrate (Thermo Fisher Scientific, cat. #34577) and imaged on the Bio-Rad ChemiDoc Touch Imaging System. Dilution and catalog numbers for primary antibodies were as follows: β-actin mouse monoclonal antibody (AC-15) (Thermo Fisher Scientific, cat. #MA1-91399, RRID: AB_2273656), P4HA1 rabbit polyclonal antibody (Thermo Fisher Scientific, cat. #PA5-52454, RRID: AB_2645146), vimentin (D21H3) XP rabbit mAb (Cell Signaling Technology, cat. #5741, RRID: AB_10695459, 1:1,000), fibronectin/FN1 (E5H6X) rabbit mAb (Cell Signaling Technology, cat. #26836, RRID: AB_2924220, 1:1,000), N-cadherin (13A9) mouse mAb (Cell Signaling Technology, cat. #14215, RRID: AB_2798427, 1:1,000), and E-cadherin (24E10) rabbit mAb (Cell Signaling Technology, cat. #3195, RRID: AB_2291471, 1:1,000). Secondary antibodies were as follows: Goat anti–mouse IgG1 secondary antibody, HRP (Thermo Fisher Scientific, cat. #PA1-74421, RRID: AB_10988195) and goat anti-rabbit IgG H&L (HRP; Abcam, cat. #ab6721, RRID: AB_955447).

### qRT-PCR

Total RNA was extracted from the organoids in each dome using a Quick-RNA Miniprep Plus kit (Zymo Research, cat. #R1057) with DNase I treatment following the manufacturer’s instructions. Isolated RNA was directly reverse transcribed to cDNA with the High-Capacity RNA-to-cDNA Kit (Applied Biosystems, 4387406) as per the manufacturer’s instructions. Thereafter, using the TaqMan Universal Master Mix II (Applied Biosystems, 4440038), qRT-PCR was carried out with three separate experimental replicates for each of the following genes obtained from IDT: *P4HA1* (Hs.PT.58.40704561), *HIF1a* (Hs.PT.58.534274), *CDH2* (N-cadherin; Hs.PT.58.26024443), *CDH1* (E-cadherin; Hs.PT.58.3324071), *SNAI1* (SNAIL; Hs.PT.58.2984401), and *VIM* (Hs.PT.58.38906895). The relative mRNA expression levels were calculated using the comparative threshold cycle (dCt) method and normalized to *GAPDH* as the reference gene (IDT, Hs.PT.39a.22214836).

### Transwell invasion assay

Transwells (8 μm pore size; Corning, cat. #3464) were coated with a coating Matrigel buffer (0.01 mol/L Tris and 0.7% NaCl, with Matrigel concentration of 200–300 μg/mL). To each of the permeable transwell support, 0.1 mL of the diluted Matrigel was added and incubated for 2 hours at 37°C. Any remaining liquid was removed, and 25,000 cells were added on top of the transwell in serum-free media (150 μL). Media with chemoattractant (FBS) were added in the wells (800 μL). Cell invasion chambers were incubated overnight in the incubator, fixed, and stained with crystal violet.

### Bulk RNA-sequencing and pathway analysis

Library preparation and RNA sequencing (RNA-seq) were performed at the Single Cell & Transcriptomics Core at Johns Hopkins using the TruSeq stranded RNA-seq library kit (Illumina). Libraries were prepared using 30 to 100 ng of RNA from each culture. For every normoxic and hypoxic invasive pair, the starting RNA input was matched for library preparation. The libraries were sequenced on the Illumina NovaSeq 6000 platform (RRID: SCR_016387), with coverage of 40 to 60 million reads per sample. RNA-seq and pathway analyses were performed using the same strategy previously described (10). The human genome was obtained in FASTA format (GRCh38) from Ensembl (19) and gene set annotation in GTF format. The hisat2 indices were built from the genome index using hisat2-build from Hisat2 version 2.2.1 (20). Raw RNA-seq paired-end reads were aligned to the genome using hisat2 after trimming with TrimGalore (RRID:SCR_011847) (21). The total reads per sample ranged from 11 to 50 million and the alignment mapping rate was greater than 77%. We next used DESeq2 (22) to estimate differential gene expression between invasive and noninvasive organoids in each invasion mode from the counts generated by HTSeq (23). We used standard DESeq2 parameters to exclude genes with no reads and those with *P* values set to the nominal value of one. Additionally, we removed genes with a low read count by requiring an average of 50 reads per patient sample. The volcano plot was made with the R package EnhancedVolcano version 1.14.1 (24). We performed pathway analysis using the R package clusterProfiler version 4.4.4 and org.Hs.eg.db version 3.15.0 (25) for Kyoto Encyclopedia of Genes and Genomes (KEGG; ref. 26) and gene ontology (GO; refs. 27, 28).

### Single-cell RNA-seq analysis

Single-cell RNA-seq (scRNA-seq) data from pancreatic tumors and normal tissue were obtained from Peng and colleagues (29), with preprocessing and cell type annotation performed as described in Guinn and colleagues (30). Cells with non-zero expression of *P4HA1* were subtyped into *P4HA1*-high and -low expression based on a 75% quantile threshold among all cells with non-zero expression. The dataset was then subset to epithelial cancer cells for all downstream analyses. Differential expression analyses were performed with Seurat version 5.0 (31) using the DESeq2 negative binomial test (22). Gene set statistics were performed using a one-sided geneSetTest in LIMMA version 3.54.0 (32) using a hypoxia gene signature derived previously (33) and Hallmark gene sets from MSigDB version 2023.2 (34). For gene set statistics computed on the Hallmark gene sets, FDR adjustment was performed using the Benjamini–Hochberg correction (35). Additionally, we annotated hypoxic tumor cells by applying a module score for all cells in the Peng and colleagues (29) dataset using our hypoxia gene set from Ye and colleagues (33). Additional analyses on hypoxic tumor cells were performed by subsetting to epithelial cancer cells with non-zero expression of *P4HA1* and having positive values of the resulting module score, with differential expression analyses performed as described previously.

### Statistics

Statistical analyses comparing the clinical and pathologic features of tumors, percentage invasion of organoids, and organoid inverse circularity scores were conducted using GraphPad PRISM (RRID: SCR_002798).

### Data availability

RNA-seq data from a subset of patients in this study will be publicly available in the Database of Genotypes and Phenotypes (dbGAP) at phs003961.v1.p1 as permitted by the Institutional Review Board based on prospective consent to data sharing. The rest of the data generated in this study are available upon request from the corresponding author. Additional data analyzed in this study were the scRNA-seq data from human PDAC tumors accessed through the Genome Sequence Archive (https://ngdc.cncb.ac.cn/bioproject/browse/PRJCA001063 GSA CRA001160), bulk RNA-seq data from primary PDAC tumors and metastases through the European Genome-phenome Archive (https://ega-archive.org/ dataset IDs EGAD00001004548 and EGAD00001003945), the KEGG and GO databases through Bioconductor (https://bioconductor.org/ package clusterProfiler version 4.4.4 and org.Hs.eg.db version 3.15.0), the Clinical Proteomic Tumor Analysis Consortium dataset through the University of Alabama at Birmingham Cancer data analysis Portal (https://ualcan.path.uab.edu/index.html), and The Cancer Genome Atlas (TCGA) dataset for pancreatic cancer through Kaplan–Meier Plotter (https://server2.kmplot.com/).

## Results

### Hypoxia promotes EMT-independent invasion in patient-derived PDAC organoids

**Figure 1:**
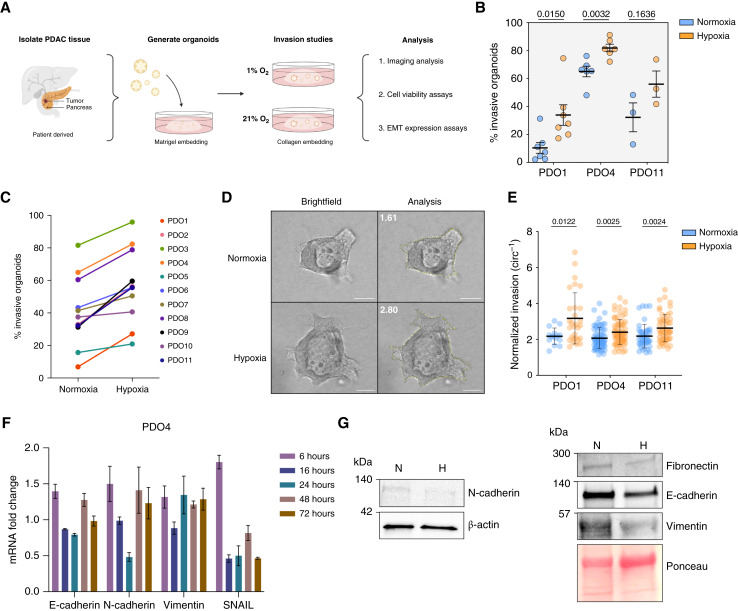
Hypoxia increases invasion in patient-derived PDAC organoids. **A,** Schematic of PDAC organoid culture generation, collection, study, and analysis timeline. **B,** Effect of hypoxia on invasion in PDO1, PDO4, and PDO11 organoids. Percent invasion represents a mean of minimum three biological replicates with SD. Each biological replicate *n* > 30 organoids (*P* = 0.015, *P* = 0.0032, and *P* = 0.1636, respectively, *t* test). **C,** Fold change of PDAC PDO sample invasion in normoxia and hypoxia. **D,** Representative images of PDO4 invasive organoids in normoxia and hypoxia. Scale bars, 100 μm. **E,** Inverse circularity scores of normoxic and hypoxic PDO1 (*n* = 15 and *n* = 29, respectively; *P* = 0.0122, *t* test), PDO4 (both *n* = 71; *P* = 0.0025, *t* test), and PDO11 (*n* = 44 and *n* = 54, respectively; *P* = 0.0024, *t* test) invasive organoids. **F,** Expression level of EMT markers (E-cadherin, N-cadherin, vimentin, and SNAIL) in PDO4 measured by qRT-PCR at multiple time points. Fold changes, expressed relative to normoxic control samples, represent a mean of three technical replicates with SD. **G,** Western blot analysis of EMT markers (fibronectin, E-cadherin, N-cadherin, and vimentin) in PDO4 in normoxia and hypoxia. H, hypoxia; N, normoxia.

To interrogate the effect of hypoxia in PDAC organoid invasion, we performed quantitative assessment of invasion in 11 human PDAC PDOs embedded in collagen I under 20% or 1% O_2_ conditions. PDOs were isolated from freshly resected human PDACs and embedded in Matrigel for passaging in normoxia (Fig. 1A; Supplementary Table S1), as previously reported (9). We have previously demonstrated that this method derives three-dimensional cultures of PDAC cells without associated non-neoplastic cells (9). Importantly, we have previously shown that phenotypes in our organoid model are associated with features in the primary tumor, including morphologic and protein expression features (9). For the current study, only PDO cultures which expanded within the first 2 weeks after Matrigel embedding were kept in culture for long-term growth and experimental use. Primary human PDOs were expanded to obtain sufficient organoids for triplicate analysis under multiple conditions. After expansion (passage < 15), PDOs were embedded in collagen I gels, and organoid invasion was assessed consistently within each culture using time-lapse microscopy for 72 to 96 hours after being placed in normoxia and hypoxia (1% O_2_), followed by image analysis (Fig. 1A). Organoids were classified as invasive or noninvasive based on the presence of either single cells or a cluster of leading cells invading into the collagen I gel in invasive organoids (Supplementary Fig. S1A). Invasion was calculated by dividing the total number of invasive organoids by the total number of organoids analyzed, hereafter referred to as the percentage of invasive organoids. Hypoxia increased the percentage of invasive organoids of all patients in our cohort [Fig. 1B and C; Supplementary Fig. S1B; Supplementary Table S2; Supplementary Videos S1 and S2; *t* test, *P* = 0.015 (PDO1); *P* = 0.0032 (PDO4); and *P* = 0.1636 (PDO11)]. PDO1, PDO4, and PDO11 patient samples expanded in culture grew more quickly than other PDOs; thus, they were used for many of the subsequent experiments. Previously, we described two morphologically defined patterns of invasion in PDAC organoids, embedded in collagen I gels: mesenchymal and collective, characterized by dissemination as single cells with an elongated spindle morphology or as cohesive multicellular units, respectively (9). PDO1 was the only organoid sample with predominantly mesenchymal invasion (>50% organoids with mesenchymal invasion, Supplementary Fig. S1A), which allowed for the exploration of invasive pattern impact on hypoxia effects. Our results show an increase in the percentage of invasive organoids in hypoxia in both patterns of invasion although the magnitude of the increase varied between samples. We observed an important consistency on the impact of hypoxia on increased percentage of invasive organoids, but some of the PDOs had a high percentage of invasion under normoxia, limiting the maximum fold change increase when in hypoxic conditions (Fig. 1C). Importantly, we did not observe a change in the pattern of invasion of any PDO cultures when in hypoxic conditions.

To quantitatively compare the degree of invasion between invasive PDOs in normoxia and hypoxia, we traced the borders of invasive organoids from PDO1, PDO4, and PDO11 to calculate an inverse circularity score, defined as 1/circularity. A high inverse circularity score indicates a higher deviation from a perfectly round organoid (noninvasive, value = 1) and can be used as a metric of how invasive an organoid is, hereafter referred to as invasiveness (36). Importantly, we observed an increase in the invasiveness of invasive organoids in hypoxia compared with organoids from the same patient in normoxia, suggesting that low-oxygen conditions can not only activate pathways to induce invasion in noninvasive organoids but can also promote a more extensive invasion in PDOs already exhibiting invasive potential (Fig. 1D and E).

We next investigated whether this hypoxia-induced invasion increase was dependent on EMT. In some studies, hypoxia has been shown to induce EMT in various cancers, which leads to functional changes in cell migration and invasion (37–39). We performed qPCR for epithelial (E-cadherin) and mesenchymal (N-cadherin, vimentin, and SNAIL) markers on PDO4 and PDO11 embedded in collagen I gels and grown in normoxia or hypoxia for 6, 16, 24, 48, and 72 hours. The transcriptional expression of epithelial and mesenchymal markers varied greatly depending on the sample and time in culture but did not follow a consistent trend. In hypoxia, most samples showed a slight decrease in E-cadherin but failed to show a significant increase in mesenchymal markers and transcriptional factors such as SNAIL (Fig. 1F; Supplementary Fig. S1C). We sought to interrogate EMT markers at the protein level by performing Western blotting for EMT markers using PDO4 organoids embedded in collagen. Interestingly, we observed a decrease of E-cadherin protein expression levels only in hypoxic organoids, whereas mesenchymal markers (vimentin, N-cadherin, and fibronectin) showed no change or a slight decrease on expression, suggesting that there is no clear EMT pathway activation at the transcriptional or protein level (Fig. 1G). To demonstrate that EMT can be induced in these PDOs under our current *in vitro* setting, we performed qPCR for epithelial (E-cadherin) and mesenchymal (N-cadherin, vimentin, and SNAIL) markers after treating organoids from PDO4 and PDO6 with 5 ng/mL TGF-β1, a well-described inducer of EMT in PDAC (40). Importantly, our results showed that TGF-β treatment was able to induce an increase in EMT markers after 72 hours (Supplementary Fig. S1D). Vimentin and N-cadherin increased significantly in both PDO4 (fold change = 8.59 and 388.25, respectively) and PDO6 organoids (fold change = 24.39 and 2.47, respectively), whereas E-cadherin decreased in organoids for both PDO4 (fold change = −2.735) and PDO6 (fold change = −416.65). Thus, these results confirm the ability of PDOs to undergo EMT in our system and suggest that hypoxic conditions are not sufficient to induce EMT in human PDAC PDOs.

To further confirm these results at the protein level, we performed IHC in paraffin-embedded collagen I domes with PDOs in normoxia and hypoxia, which showed no expression of the mesenchymal marker vimentin and consistent expression of the epithelial marker E-cadherin (Supplementary Fig. S1E). These results further support that hypoxia can increase invasion in PDAC PDOs in an EMT-independent manner. In addition, these spatially resolved analyses confirm that EMT-associated changes are not limited to organoid invasive protrusions as these structures showed an intact expression of E-cadherin and no expression of vimentin.

In most cell types, hypoxia decreases cell proliferation, but in certain cell populations, cell proliferation might be maintained, even under hypoxic stress (41). We therefore sought to assess the impact of hypoxic conditions on cell proliferation in PDAC PDOs. We performed a CellTiter-Glo assay using five PDAC PDOs embedded in collagen I and grown in normoxia and hypoxia for several days. PDOs were selected based on their ability to proliferate and expand in Matrigel. Overall, differences in the proliferation of PDOs in normoxia and hypoxia were nonsignificant (Supplementary Fig. S1F). Due to the use of Matrigel to expand our PDAC PDOs, we also performed the same assay using PDO1 and PDO3 organoids embedded in Matrigel to assess whether the type of ECM affected the growth of the organoids. In Matrigel, PDO1 and PDO3 organoids grew significantly slower in hypoxia compared with normoxia at most timepoints (Supplementary Fig. S1G). These results show that the type of extracellular matrix (ECM) used for embedding PDAC PDOs influences the impact of hypoxia on cell proliferation.

Overall, these results suggest that hypoxia is a mediator of EMT-independent invasion in PDAC PDOs, by both increasing the degree of invasiveness as well as the percentage of invasive organoids embedded in collagen I gels.

### Hypoxia upregulates P4HA1 expression in PDAC PDOs through HIF1α

Having observed an increase in the invasiveness and percentage of invasive organoids grown in hypoxia, we sought to identify the transcriptomic signatures associated with invasion in hypoxic PDAC PDOs by performing RNA-seq. To do this, we used a method we recently developed to separate organoids within the same collagen I dome, in which only organoids exhibiting invasive features are manually isolated (10, 42). Using this method, organoid cultures from a total of eight PDACs were sorted to isolate only invasive organoids from normoxic and hypoxic conditions for subsequent RNA-seq. This allowed us to interrogate the differences observed because of hypoxic conditions and distinguish these differences from those broadly related to enhancement of invasion across conditions.

**Figure 2:**
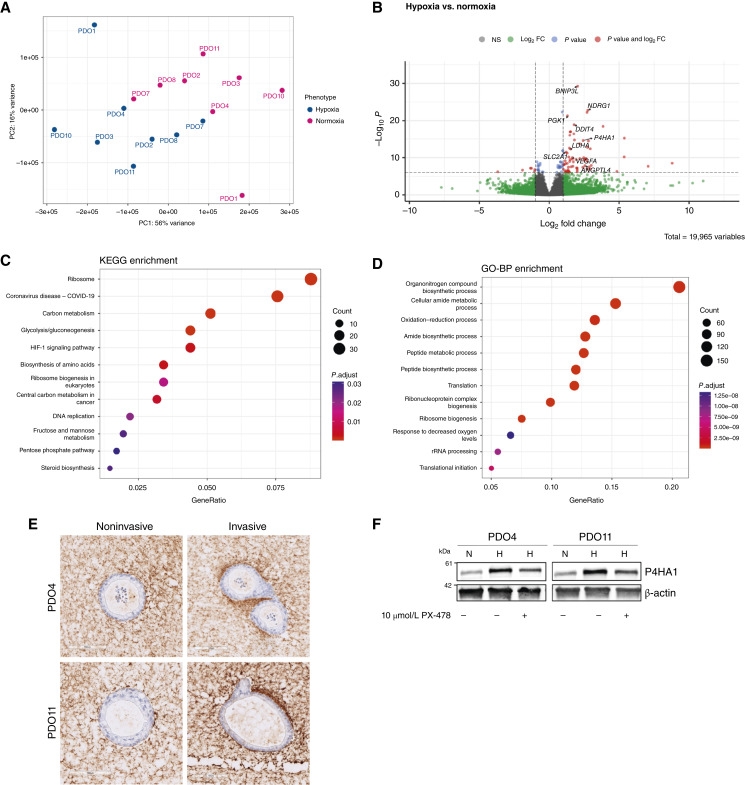
Comparison of transcriptomes and molecular pathways of invasive normoxic and hypoxic organoids. **A,** Principal component analysis plot of gene expression in hypoxic invasive organoid samples (*n* = 8). PC, principal component. **B,** Volcano plot of differentially expressed genes between normoxic and hypoxic invasive organoids. FC, fold change. **C,** Pathway analysis of normoxic and hypoxic invasive organoids using KEGG processes. **D,** Pathway analysis of normoxic and hypoxic invasive organoids using GO Biological Processes (GO-BP). **E,** IHC for hydroxyproline surrounding invasive and noninvasive organoids in PDO4 and PDO11. **F,** Western blot analysis assessing P4HA1 levels in 2D cultures prepared from PDO4 and PDO11 organoids 24 hours after treatment with a HIF1a inhibitor (10 μmol/L PX-478). H, hypoxia; N, normoxia.

To identify the transcriptomic alterations associated with invasion in hypoxia, we performed differential gene expression analysis of paired normoxic and hypoxic invasive organoid cultures derived from eight primary PDACs. Principal component analysis showed that the first and second principal components separated the normoxic and hypoxic organoids (Fig. 2A). The differential gene expression analysis identified 875 genes that were differentially expressed between normoxic and hypoxic invasive organoids (FDR < 0.05; Fig. 2B; Supplementary Table S3). Notably, among the 875 genes, 230 genes were upregulated in the hypoxic organoids. Importantly, we identified many previously defined hypoxia-regulated genes (Supplementary Table S4), encompassing pathways related to cell metabolism (LDHA, SLC2A1, and PGK1), cell growth and apoptosis (DDIT4), angiogenesis (VEGFA and ANGPTL4), and ECM remodeling (P4HA1; Fig. 2B). We next performed pathway analysis of KEGG and GO Biological Processes using differentially expressed genes between invasive organoids in normoxia and hypoxia (Fig. 2C and D; Supplementary Fig. S2A). As expected, the KEGG and GO Biological Process enrichments revealed that invasive hypoxic organoids have differentially expressed genes mostly related to metabolism, response to decreasing oxygen levels, and HIF1α-regulated pathways. Pathway analysis did not reveal an upregulation of EMT pathways, supporting our initial results at the transcriptomic and protein levels (Fig. 1F and G).

Because of its role in collagen deposition and correct folding of collagen fibers (13), we explored the role of P4HA1 as a potential modulator of invasion in PDAC PDOs. First, we performed hematoxylin and eosin staining on sections and IHC from formalin‐fixed, paraffin-embedded invasive and noninvasive PDAC organoids in collagen I to demonstrate the morphology and epithelial origin of generated organoids (Supplementary Fig. S2B). Then, to determine the association of collagen modification with cancer cell invasion, we assessed hydroxyproline via IHC in the collagen surrounding both noninvasive and invasive PDAC PDOs in normoxia. P4HA1 acts to modify collagen, later deposited in the ECM, and therefore localized increase in hydroxylated collagen surrounding the organoids is expected. We observed this increased hydroxyproline in the collagen surrounding organoids, especially around invasive protrusions, suggesting that collagen modification is associated with organoid invasion (Fig. 2E). As P4HA1 expression was increased by hypoxia in our system, we interrogated whether hypoxia-induced P4HA1 overexpression was dependent on HIF1α by treating two-dimensional (2D) cultures of PDAC cells prepared from PDO4 and PDO11 with the HIF1α inhibitor PX-478 while in hypoxia. Our results show that the treatment of PDO4 and PDO11 cells with 10 μmol/L of PX-478 for 24 hours reduced the levels of P4HA1 in hypoxia (Fig. 2F), suggesting P4HA1 expression in hypoxia is regulated by HIF1α expression.

Next, we explored whether the impact of P4HA1 on invasion was limited to hypoxic conditions. Because of the heterogeneity in the basal percentage invasion of PDOs in normoxia (Fig. 1C), we first interrogated whether these invasion differences were associated with differential P4HA1 expression levels. Specifically, in our invasive organoid RNA-seq data, we explored the correlation between percentage invasion with VST^−1^-transformed P4HA1 expression. Analysis of normoxic PDO samples showed no correlation between P4HA1 expression levels and percentage invasion of organoids (Supplementary Fig. S2C). Overall, these results suggest that the heterogeneous invasion of PDAC PDOs in normoxia is likely driven by other molecular programs, such as those reported recently, which identified differentially expressed genes associated with invasion (10). P4HA1 was not significantly differentially expressed between invasive and noninvasive normoxic organoids in this dataset (fold change = 0.211; *P* = 0.419), suggesting that elevated P4HA1 activity may be a feature but not a major driver of invasion in normoxia (Supplementary Fig. S2D). Taken together, these results support that the impact of P4HA1 in enhancing invasion is limited to hypoxic conditions.

Our RNA-seq analysis only included invasive organoids in normoxic and hypoxic conditions; thus, we performed qPCR to assess the expression of P4HA1 in noninvasive and invasive organoids grown in normoxic and hypoxic conditions isolated from three PDOs. P4HA1 was upregulated in organoids grown in hypoxia but no significant differences were observed in invasive hypoxic organoids compared with noninvasive hypoxic ones, suggesting that P4HA1 is a hypoxia-induced gene (Supplementary Fig. S2E).

Given these results using our organoid model, we sought to further investigate the role of P4HA1 in PDAC tissue datasets.

### P4HA1 expression is elevated in PDAC and correlates with hypoxia and patient outcomes

**Figure 3:**
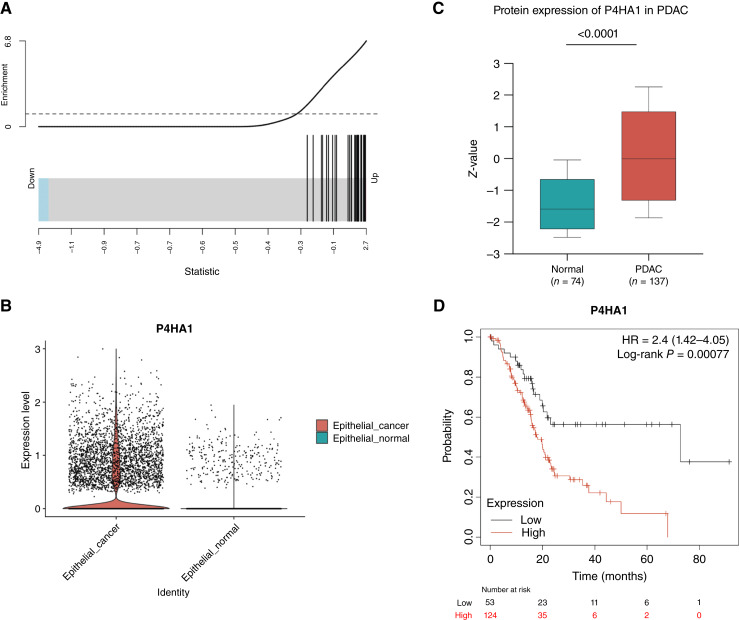
P4HA1 expression is increased in PDAC and is clinically relevant. **A,** Gene set enrichment analysis showing a correlation between a high P4HA1 expression and the expression of genes in a hypoxia gene signature (*P* = 3 × 10^−20^). **B,** scRNA-seq analysis shows increased expression levels of P4HA1 in human pancreatic tumors compared with normal tissue (*P* = 9 × 10^−16^). **C,** Protein expression of P4HA1 in PDAC using the Clinical Proteomic Tumor Analysis Consortium database, accessed through the University of Alabama at Birmingham Cancer data analysis Portal. **D,** Kaplan–Meier analysis of patients with PDAC shows increased risk of death associated with a high expression of P4HA1. Plot generated using KMPlot. The median survival time for low expression and high expression cohorts is 72.73 and 18.17 months, respectively (HR = 2.4; 95% confidence interval, 1.42–4.05; *P* = 0.00077).

We explored the gene and protein expressions of P4HA1 in normal pancreas and PDAC tissues using publicly available datasets. We first confirmed the correlation of P4HA1 with hypoxia in tumor cells from primary human PDAC tissue using scRNA-seq data. Using the scRNA-seq data from primary human PDAC tissue reported in Peng and colleagues (29), we isolated tumor cells using the cell type labels annotated as described in Guinn and colleagues (30). We sought to compare the gene expression profiles between tumor cells that were in the top 75% quantile of P4HA1 expression relative to all other tumor cells with non-zero expression of P4HA1, thereby minimizing the impact of drop-out of the single-cell assay on inference of molecular signaling. Gene set enrichment analysis demonstrated that a previously validated hypoxia gene signature (33) was significantly enriched in cells with high P4HA1 expression (Fig. 3A, *P* = 3 × 10^−20^), confirming that P4HA1 upregulation in hypoxia is not specific to our organoid model and also occurs in human PDAC tissue samples. In the same scRNA-seq dataset, we also assessed P4HA1 gene expression in PDAC tumor cells enriched in the hypoxia gene signature, further confirming the association of P4HA1 expression and hypoxia (Supplementary Fig. S3A; fold change = 2.073, *P* = 4.188 × 10^−126^). Then, we analyzed expression of P4HA1 in primary human normal epithelial and PDAC cells, demonstrating higher levels of P4HA1 expression in PDAC cells compared with normal pancreatic epithelial cells (Fig. 3B). In addition, using a separate bulk RNA-seq dataset from human PDAC samples, we compared P4HA1 expression in primary and metastatic PDACs (43). The expression of P4HA1 was increased in metastases although not significantly (Supplementary Fig. S3B, *P* = 0.1337). However, these results should be interpreted with caution – although increased P4HA1 expression in metastases could reflect the increased invasiveness, the results could also be influenced by the differential oxygenation of primary versus metastatic sites.

Next, we analyzed bulk proteomic and transcriptomic PDAC datasets to further assess the association of P4HA1 expression with clinical variables. The Clinical Proteomic Tumor Analysis Consortium dataset, accessed using the University of Alabama at Birmingham Cancer data analysis Portal (44–46) which includes protein expression data from 74 human normal pancreas and 137 human PDAC samples, showed that P4HA1 protein expression is upregulated in PDAC (*n* = 137) compared with normal pancreas (*n* = 74; Fig. 3C, *t* test *P* < 0.0001). Using the KMplot portal, we obtained information on normal and PDAC samples from TCGA and investigated the impact of P4HA1 on overall survival (47, 48). A Kaplan–Meier analysis of TCGA PDAC cohort identified that the increased expression of P4HA1 was associated with worse overall survival (Fig. 3D, *P* = 0.00077). Other hypoxia-regulated genes identified in our differential gene expression analysis, such as VEGFA and NDRG1, showed increased protein levels compared with normal pancreatic tissue (Supplementary Fig. S3C and S3E) but did not have prognostic value on overall survival, suggesting that this effect is specific to P4HA1 and not solely because of hypoxia (Supplementary Fig. S3D and S3F).

Given our results in PDAC tissue datasets confirming the association of P4HA1 expression with hypoxia and clinical outcomes, we next sought to interrogate its impact on invasion by molecularly modifying its expression in our PDAC PDO model.

### P4HA1 is required for hypoxia-induced invasion in PDAC PDOs

**Figure 4:**
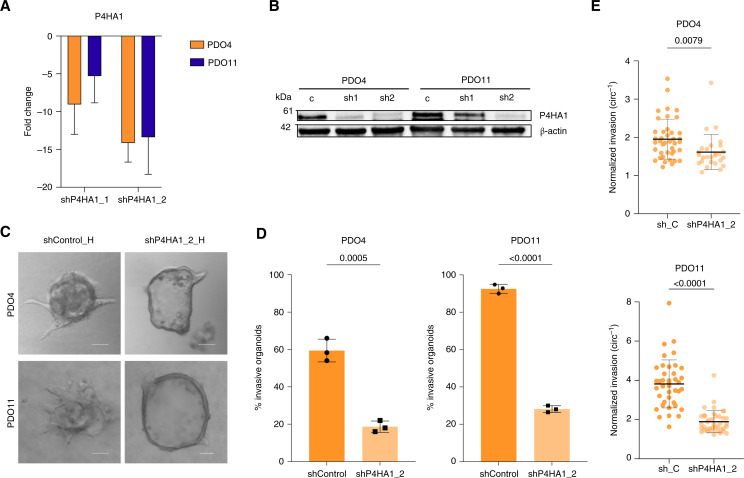
Knockdown of P4HA1 decreases invasion in PDAC PDOs in hypoxia. **A,** Expression level of P4HA1 measured by qRT-PCR. Fold changes, expressed relative to the modified control samples, represent a mean of three technical replicates with SDs (PDO4 *P* = 0.0547, *t* test; PDO6 *P* = 0.0816, *t* test). **B,** Western blot analysis to confirm effectiveness of P4HA1 knockdown in PDO4 and PDO11 compared with the empty vector. **C,** Representative images of invasive shControl and shP4HA1_2-modified organoids in hypoxia. Scale bars, 100 μm. **D,** Effect of P4HA1 knockdown on invasion in modified PDO4 and PDO11 organoids. Percent invasion represents a mean of three biological replicates with SDs. Each biological replicate *n* > 30 organoids. (*P* = 0.0005 and *P* < 0.0001, *t* test). **E,** Inverse circularity scores of empty vector–modified and P4HA1 knockdown–modified PDO4 (top; *n* = 41 and *n* = 27, respectively; *P* = 0.0079, *t* test) and PDO11 (down; both *n* = 40; *P* < 0.0001, *t* test) organoids in hypoxia.

We next sought to investigate the requirement of P4HA1 for invasion under hypoxic conditions. We knocked down P4HA1 in two PDAC PDOs (PDO4 and PDO11) using lentiviral vector–mediated transduction. We used two different shRNA constructs to determine their efficiency at decreasing P4HA1 expression in PDAC PDOs. After puromycin selection, P4HA1 knockdown was confirmed by qPCR (Fig. 4A) and Western blotting (Fig. 4B), with the shP4HA1_2 construct being the most effective at decreasing P4HA1 expression both at the RNA and protein levels (Fig. 4B). After expansion of modified organoids for experimental analysis, we observed that PDO11 organoids transduced with the shP4HA1_1 construct slowed down their growth in culture, which was confirmed using a CellTiter-Glo assay in organoids embedded in Matrigel (Supplementary Fig. S4A), thus impeding our ability to expand them significantly for use in further experiments. Because of this, we assessed the role of P4HA1 in invasion using the shP4HA1_2 construct in both organoid lines, whereas the shP4HA1_1 construct was only used for PDO4 organoids.

To determine the requirement for P4HA1 in hypoxia-induced invasion in PDAC organoids, we embedded modified control and knockdown PDO4 and PDO11 organoids in collagen I gels, and organoid invasion was assessed using time-lapse microscopy for 72 to 96 hours in hypoxia, followed by image analysis (Fig. 4C). Imaging analysis of invasive organoids showed a decrease in invasiveness in the organoids with P4HA1 knockdown (Fig. 4C; Supplementary Videos S3 and S4). Importantly, there was a decrease in the percentage of invasive organoids in both PDO4 and PDO11 when P4HA1 was knocked down in hypoxic conditions (*P* = 0.0005 and *P* < 0.0001, respectively; Fig. 4D; Supplementary Fig. S4B). We also observed a decrease in the invasiveness of invasive organoids (as measured by organoid circularity) in both PDO4 and PDO11 with P4HA1 knockdown compared with control organoids (Fig. 4E; *P* = 0.0079 and *P* < 0.0001, respectively; Supplementary Fig. S4C). Consistent with our prior findings in our organoid model, PDAC PDO shControl organoids showed an increase in the percentage of invasive organoids in hypoxic conditions, confirming that organoid transduction did not affect the hypoxia-dependent increase in invasion (Supplementary Fig. S4D). To determine whether hypoxic conditions are needed for P4HA1 to modulate invasion in PDAC PDOs, we performed the same invasion experiments in normoxia. Interestingly, neither PDO4 nor PDO11 showed a significant decrease in the percentage of invasive organoids in the P4HA1 knockdown condition when cultured in normoxia (Supplementary Fig. S4E and S4F), suggesting that a hypoxic environment may be needed for P4HA1 to play a role in invasion. However, knockdown of P4HA1 in normoxia significantly decreased the invasiveness of invasive PDO11 organoids (*P* < 0.0001), whereas no significant decrease was observed for PDO4 organoids (*P* = 0.2717). This suggests that the requirement for P4HA1 in invasion in normoxia could be variable between patients (Supplementary Fig. S4G; Supplementary Videos S5 and S6).

Overall, these results identify P4HA1 as a key regulator of invasion in PDAC PDOs in hypoxia.

### Overexpression of P4HA1 increases invasiveness in PDAC PDOs in normoxia

**Figure 5:**
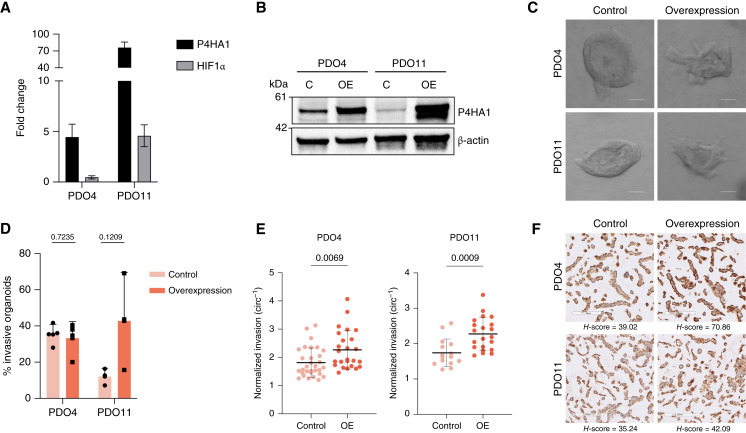
Overexpression of P4HA1 increases invasiveness in PDAC PDOs in normoxia. **A,** Expression level of P4HA1 measured by qRT-PCR. Fold changes, expressed relative to the modified control samples, represent a mean of three technical replicates with SDs. **B,** Western blot analysis to confirm the effectiveness of P4HA1 overexpression in PDO4 and PDO11 compared with the empty vector. **C,** Representative images of invasive empty vector–modified and overexpressed P4HA1–modified organoids in normoxia. Scale bars, 100 μm. **D,** Effect of P4HA1 overexpression on invasion in modified PDO4 and PDO11 organoids. Percent invasion represents a mean of three biological replicates with SDs. Each biological replicate *n* > 30 organoids (*P* = 0.7235 and *P* = 0.1209, *t* test). **E,** Inverse circularity scores of empty vector–modified and P4HA1 overexpression–modified PDO4 (*n* = 31 and *n* = 25, respectively; *P* = 0.0069, *t* test) and PDO11 (*n* = 15 and *n* = 21, respectively; *P* = 0.0009, *t* test) organoids in normoxia. **F,** IHC for hydroxyproline in HistoGel-embedded cell pellets prepared from 2D cultures of PDO4 and PDO11 P4HA1 overexpression and control lines. C, control; OE, overexpression.

Next, we sought to determine the effect of P4HA1 independently of other molecular alterations induced by hypoxia. We overexpressed P4HA1 in two PDAC PDOs (PDO4 and PDO11) using lentiviral vector–mediated transduction. After puromycin selection, P4HA1 overexpression was confirmed by qPCR and Western blotting (Fig. 5A and B).

Because P4HA1 is overexpressed in invasive PDAC organoids in hypoxia, we sought to determine whether P4HA1 overexpression in normoxia would recapitulate the enhanced invasion seen in hypoxia. We embedded control and P4HA1-modified PDO4 and PDO11 organoids in collagen I gels, and organoid invasion was assessed using time-lapse microscopy for 72 to 96 hours in normoxia, followed by image analysis (Fig. 5C; Supplementary Videos S7 and S8). We did not observe a significant increase in the percentage of invasive organoids when P4HA1 was overexpressed (Fig. 5D; *P* = 0.7235 and *P* = 0.1209, respectively). Importantly, we observed an increase in the invasiveness of invasive organoids in both PDO4 and PDO11 with overexpressed P4HA1 compared with control organoids (Fig. 5E; *P* = 0.0069 and *P* = 0.0009, respectively).

Considering the function of P4HA1 as a collagen-modifying enzyme, we sought to determine whether the impact of P4HA1 on invasion was limited to assays in collagen matrices. To quantify invasion in a transwell Matrigel assay, we first established monolayer cultures from control and P4HA1-overexpressing PDO4 and PDO11 cells. We confirmed selective growth of transduced cells by detection of eGFP and mCherry fluorescent markers (control and P4HA1 overexpression construct, respectively) on PDO4 monolayer cultures (Supplementary Fig. S5A). We then assessed the role of P4HA1 in a transwell Matrigel invasion assay. P4HA1 overexpression significantly increased the number of invading single cells in both PDO4 (fold change = 1.29; *P* = 0.0114, *t* test) and PDO11 (fold change = 1.81; *P* = 0.0479, *t* test) cultures compared with the transduced control (Supplementary Fig. S5B and S5C). These results suggest that the role of P4HA1 in enhancing invasion is not limited to pure collagen matrices although it is important to note that collagen is also a component of Matrigel (49). Finally, we confirmed that P4HA1 overexpression is sufficient to increase the level of hydroxyproline in our *in vitro* models. We performed IHC for hydroxyproline, followed by image quantification using the HALO pipeline (RRID: SRC_018350), which showed elevated levels in 2D cultures prepared from PDO4 (H-score = 39.02 and 70.86; control and overexpression, respectively) and PDO11 (H-score = 35.24 and 42.09; control and overexpression, respectively) in which P4HA1 was overexpressed (Fig. 5F).

Overall, these results in normoxic cultures suggest that P4HA1 can enhance invasion in the absence of other molecular changes induced by hypoxia, confirming its role as a key mediator of hypoxia-induced invasion in PDAC PDOs.

## Discussion

Through our work, we have shown that hypoxic conditions consistently increase the percentage of invasive organoids in a sizable cohort of 11 human PDAC PDOs in an EMT-independent manner and identified P4HA1 overexpression as necessary for hypoxia-enhanced PDAC invasion. The overexpression of P4HA1 increased the invasiveness of PDOs in normoxia, and knockdown of P4HA1 significantly diminished the effect of hypoxia on invasion. Taken together, our results suggest that hypoxia induces PDAC invasion through the increased expression of P4HA1.

To our knowledge, this study is the first study to employ PDAC PDOs to interrogate the effects of hypoxia on invasion and to determine key mediators driving invasion in hypoxia. Hypoxia consistently increased the percentage of invasion across all PDAC PDOs in our cohort, irrespective of their initial invasive pattern or level of invasion in normoxia (Fig. 1C). In many solid tumors, including PDAC, hypoxia is a well-established biological phenomenon due to desmoplastic stroma, aberrant cell proliferation, altered cellular metabolism, and formation of abnormal tumor blood vessels (50). Importantly, although PDAC studies have highlighted the association of hypoxia with tumor progression, cell migration, ECM degradation, and angiogenesis (50–53), our study provides additional important insights on hypoxia-driven invasion as it uses primary low-passage cultures of human PDOs, which more closely recapitulate native PDAC biology. Additionally, our results show a consistent increase in invasion under hypoxia in a sizable patient cohort and our model allows for the quantification of relevant invasive phenotypes.

In cell culture and mouse models, previous studies have reported that PDAC cells in hypoxia exhibit a decreased expression of epithelial markers and increased mesenchymal ones, suggesting activation of EMT (38, 54, 55). Hypoxia-induced EMT has also been shown in other cancer types such as in colorectal, hepatocellular, and breast, among others (37, 39, 56). Interestingly, our results challenge the conventional understanding surrounding hypoxia-driven EMT-mediated invasion. Our transcriptional and protein-level data (Fig. 1F and G) show that hypoxia maintains, rather than decreases, epithelial characteristics and suggest that there may be distinct hypoxia-driven invasive programs or molecular pathways independent of EMT activation. This was confirmed in our RNA-seq and pathway analysis, which did not show EMT pathways being increased in PDAC PDOs in hypoxia. Importantly, we previously described two patterns of invasion and showed that collective and mesenchymal invasive phenotypes correlated with the expression of epithelial and mesenchymal markers, respectively (9). In our cohort, hypoxia increased invasion in both phenotypes and did not cause a change of invasive pattern phenotype in any of our patient cohort samples. As hypoxia had negligible influence on proliferation in our system, our results suggest that hypoxia orchestrates discrete effects on invasion-related processes and raises the need to identify signaling pathways mediating these effects, irrespective of EMT activation.

Because of the association of hypoxia with many cellular processes and clinical relevance, recent studies have focused on determining hypoxia gene signatures to predict clinical outcomes and response to therapy (57–59). Our PDAC PDO RNA-seq results in normoxia and hypoxia showed more than 800 differentially expressed genes related to metabolism, cell growth, and angiogenesis, among others (Fig. 2); these data could help refine a PDAC-specific hypoxia signature. The upregulation of P4HA1 under hypoxia, which we confirmed in human PDAC tissue using publicly available scRNA-seq data, emerged as a key potential driver of hypoxia-induced invasion in PDAC because of its role in ECM deposition and remodeling (60). In line with this, recent studies have associated P4HA1 with clinically important cancer phenotypes, with correlation of P4HA1 expression with cancer stage and poor prognosis in breast and lung cancers, among others (17, 61). This raises the possibility of a common thread linking P4HA1 to invasion across diverse malignancies and suggests that targeting P4HA1 may hold promise across multiple cancer types.

Our results show that P4HA1 overexpression is sufficient to increase PDAC invasiveness in normoxia and that P4HA1 expression is necessary for PDAC invasion only in hypoxia. Previous studies have reported similar observations about the role of P4HA1 in promoting invasion in different cancer types, including glioblastoma and colorectal cancer (62, 63). However, most previous studies exploring the impact of P4HA1 were performed using 2D models, with P4HA1 overexpression increasing proliferation, migration, and invasion, often relying on single-cell invasion through Matrigel as the assayed phenotype (17, 62–64). Importantly, our PDAC PDO model allows for the study of invasion in a collagen I microenvironment and while maintaining cell–cell interactions within the organoids. Intriguingly, P4HA1 overexpression resulted in a modest but significant increase in invasion in Matrigel invasion assays in our study, suggesting that the impact of P4HA1 on invasion may vary based on the amount of collagen I present in the ECM used.

Other studies have also interrogated the mechanism by which P4HA1 regulates cancer invasion in other tumor types, identifying EMT and matrix metalloproteinase activity as potential downstream mediators (65, 66). Prior to our study, the potential mechanisms by which P4HA1 increases invasion in hypoxia in PDAC were unexplored. Using hydroxyproline IHC, we demonstrate that overexpression of P4HA1 leads to increased hydroxyproline and that hydroxyproline is enriched in collagen surrounding invasive protrusions, identifying the direct downstream consequences of P4HA1 elevation. These results suggest collagen remodeling and deposition as further downstream mechanisms that should be interrogated in future studies.

In our study, we employed passaged human PDAC organoids to assess the impact of hypoxia on invasion. Although these primary human PDAC samples are a powerful system to interrogate the molecular mechanisms of invasion, this system has multiple limitations. Although we initially interrogated a cohort of 11 patient samples, our experimental approach required large numbers of organoids over the course of multiple experiments, including molecular modification of organoids over multiple passages. This requirement for expansion in culture prior to experimental manipulation limited the PDOs that could be used as not all PDAC PDOs were able to proliferate and expand at the same rate or efficiency. Importantly, our PDAC PDO model does not allow for the analysis of the molecular features of invasive protrusions in dissociative analyses such as RNA-seq. Thus, future studies should focus on developing spatially resolved molecular approaches to be able to effectively assess the molecular characteristics of those invasive protrusions. In addition, previous studies demonstrated that long-term passaging of PDAC PDOs can affect transcriptional phenotypes (67) and potentially select for specific organoid characteristics over time. The relevance of these culture-induced alterations on hypoxia-driven invasion remains unexplored. Finally, our cohort consisted of all surgically resected tumors, which now represent a minority of patients diagnosed with PDAC (1). Recent successful generation of organoid cultures using biopsies (6, 68) opens the possibility to study the impact of hypoxia and the role of P4HA1 in invasion using samples from different PDAC stages and would elucidate whether hypoxic PDAC cells can metastasize to distant organs.

In summary, our study leverages a sizable cohort of human PDAC PDOs to study the impact of hypoxia on invasion. We show that hypoxia increases invasion across all patients and identify P4HA1 as a key regulator of organoid invasion only in hypoxia. Taken together, our results provide important insights into the role of hypoxia in driving PDAC cell invasion and highlight the potential of targeting P4HA1 to inhibit invasion.

## Supplementary Material

List of Supplementary Materials List of Supplementary Materials

Supplementary Figure S1 Hypoxia increases invasion in PDAC organoids in absence of consistent EMT phenotype

Supplementary Figure S2 Hypoxia alters gene expression in PDAC organoids

Supplementary Figure S3 Hypoxia-associated genes are upregulated in PDAC but not associated with clinical prognosis

Supplementary Figure S4 Impact of P4HA1 knockdown on organoid invasion in normoxia

Supplementary Figure S5 Effect of P4HA1 overexpression on invasion of PDAC cells in Matrigel

Supplementary Table S1 Primary tumor features in PDAC organoid cohort

Supplementary Table S2 Percent invasion of organoids in normoxia and hypoxia

Supplementary Table S3 Differentially expressed genes between normoxic and hypoxic invasive PDAC organoids

Supplementary Table S4 Differential expression of hypoxic genes

Supplementary Video S1 Invasion of PDAC organoids in normoxia

Supplementary Video S2 Invasion of PDAC organoids in hypoxia

Supplementary Video S3 Time-lapse analysis of empty vector modified PDAC organoids in hypoxia

Supplementary Video S4 Time-lapse analysis of P4HA1 knockdown modified PDAC organoids in hypoxia

Supplementary Video S5 Time-lapse analysis of empty vector modified PDAC organoids in normoxia

Supplementary Video S6 Time-lapse analysis of P4HA1 knockdown modified PDAC organoids in normoxia

Supplementary Video S7 Time-lapse analysis of empty vector modified PDAC organoids in normoxia

Supplementary Video S8 Time-lapse analysis of P4HA1 overexpression modified PDAC organoids in normoxia
