# Supplementary material for: P4HA1 Mediates Hypoxia-Induced Invasion in Human Pancreatic Cancer Organoids
Source: Cancer Res Commun. 2025 May 30;5(5):881–95. doi: 10.1158/2767-9764.CRC-24-0025 (PMC12123483; doi:10.1158/2767-9764.CRC-24-0025)
Supplement: List of Supplementary Materials [file crc-24-0025_list_of_supplementary_materials_suppsm.docx]

**Supplementary Figures**

**Supplementary Figure S1.** Hypoxia increases invasion in PDAC organoids in absence of consistent EMT phenotype.

**Supplementary Figure S2.** Hypoxia alters gene expression in PDAC organoids.

**Supplementary Figure S3**. Hypoxia-associated genes are upregulated in PDAC but not associated with clinical prognosis

**Supplementary Figure S4.** Impact of P4HA1 knockdown on organoid invasion in normoxia.

**Supplementary Figure S5.** Effect of P4HA1 overexpression on invasion of PDAC cells in Matrigel.

**Supplementary Tables**

**Supplementary Table S1.** Primary tumor features in PDAC organoid cohort.

**Supplementary Table S2.** Percent invasion of organoids in normoxia and hypoxia.

**Supplementary Table S3.** Differentially expressed genes between normoxic and hypoxic invasive PDAC organoids.

**Supplementary Table S4.** Differential expression of hypoxic genes.

**Supplementary Videos**

**Supplementary Video S1.** Invasion of PDAC organoids in normoxia (scale bar: 100μm)

**Supplementary Video S2.** Invasion of PDAC organoids in hypoxia (scale bar: 100μm)

**Supplementary Video S3.** Time-lapse analysis of empty vector modified PDAC organoids in hypoxia (scale bar: 200μm)

**Supplementary Video S4.** Time-lapse analysis of P4HA1 knockdown modified PDAC organoids in hypoxia (scale bar: 200μm)

**Supplementary Video S5.** Time-lapse analysis of empty vector modified PDAC organoids in normoxia (scale bar: 100μm)

**Supplementary Video S6.** Time-lapse analysis of P4HA1 knockdown modified PDAC organoids in normoxia (scale bar: 100μm)

**Supplementary Video S7.** Time-lapse analysis of empty vector modified PDAC organoids in normoxia (scale bar: 100μm)

**Supplementary Video S8.** Time-lapse analysis of P4HA1 overexpression modified PDAC organoids in normoxia (scale bar: 100μm)
