# Supplementary figures and images for: P4HA1 Mediates Hypoxia-Induced Invasion in Human Pancreatic Cancer Organoids
Source: Cancer Res Commun. 2025 May 30;5(5):881–95. doi: 10.1158/2767-9764.CRC-24-0025 (PMC12123483; doi:10.1158/2767-9764.CRC-24-0025)

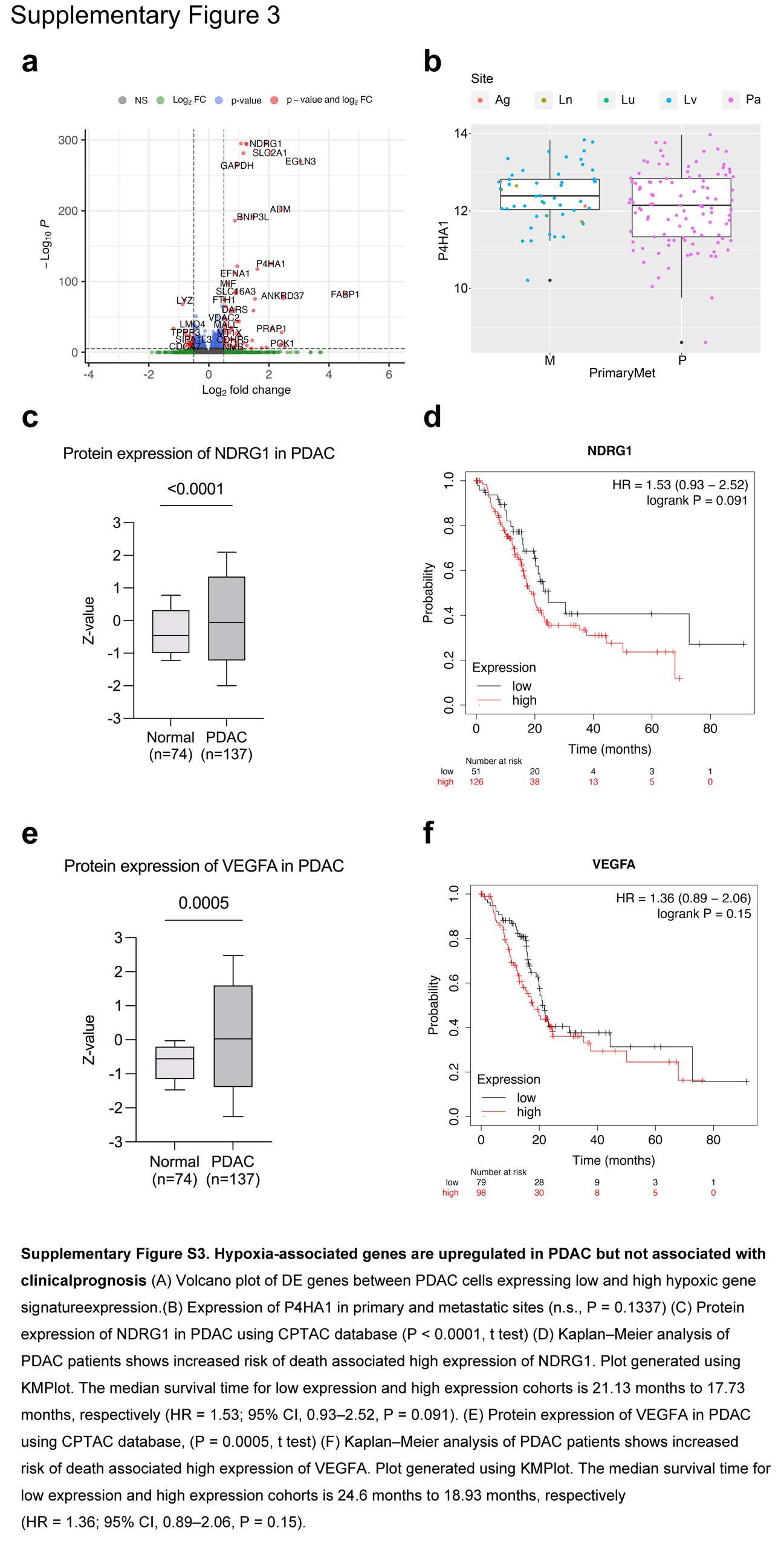

Supplement: Supplementary Figure S3 — Hypoxia-associated genes are upregulated in PDAC but not associated with clinical prognosis [file crc-24-0025_supplementary_figure_s3_suppsf3.png]

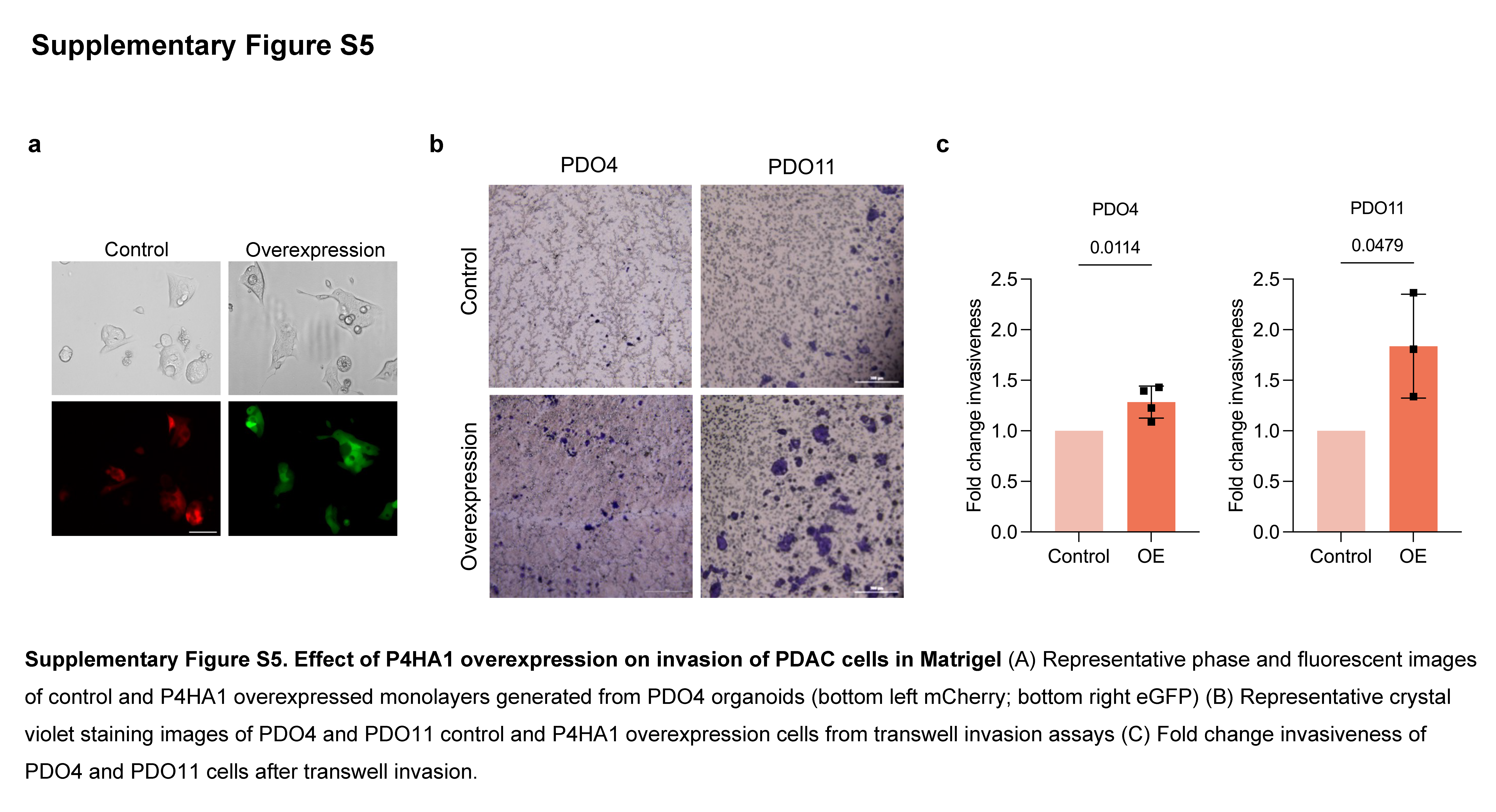

Supplement: Supplementary Figure S5 — Effect of P4HA1 overexpression on invasion of PDAC cells in Matrigel [file crc-24-0025_supplementary_figure_s5_suppsf5.png]
